# Immune Checkpoint Inhibitors as a Threat to the Hypothalamus–Pituitary Axis: A Completed Puzzle

**DOI:** 10.3390/cancers14041057

**Published:** 2022-02-18

**Authors:** Agnese Barnabei, Andrea Corsello, Rosa Maria Paragliola, Giovanni Maria Iannantuono, Luca Falzone, Salvatore Maria Corsello, Francesco Torino

**Affiliations:** 1 Endocrinology Unit, P.O.—S. Spirito in Sassia, ASL Roma 1, Lungotevere in Sassia 1, I-00193 Rome, Italy; agnese.barnabei@aslroma1.it; 2 Unit of Endocrinology, Department of Translational Medicine and Surgery, Università Cattolica del Sacro Cuore-Fondazione Policlinico “Gemelli” IRCCS, Largo Gemelli 8, I-00168 Rome, Italy; andrea.corsello01@icatt.it (A.C.); rosamaria.paragliola@unicatt.it (R.M.P.); corsello.sm@meridiaroma.it (S.M.C.); 3 Medical Oncology Unit, Department of Systems Medicine, University of Rome Tor Vergata, Via Montpellier 1, I-00133 Rome, Italy; gmiannantuono@gmail.com; 4 Epidemiology Unit, IRCCS Istituto Nazionale Tumori ‘Fondazione G. Pascale’, I-80131 Naples, Italy; l.falzone@istitutotumori.na.it; 5 UniCamillus, Saint Camillus International University of Health Sciences, Via di Sant’Alessandro, 8, I-00131 Rome, Italy

**Keywords:** immune checkpoint inhibitors, hypophysitis, central diabetes insipidus, hypothalamitis, endocrinopathy, posterior pituitary

## Abstract

**Simple Summary:**

Endocrine dysfunctions are among the most frequent toxicities induced by immune checkpoint inhibitors (ICI). Recent evidence suggests that each tract of the hypothalamic–pituitary axis can be injured by ICI, though at different frequencies. According to the limited literature, ICI agents modulating the PD-1/PD-L1 axis rarely cause posterior pituitary/hypothalamic dysfunction. In comparison, anti-CTLA4 agents seemed to affect the function of the posterior pituitary only through the extension of inflammation/autoimmunity reactions primarily triggered in the anterior pituitary (panhypophysitis). Waiting for more extensive data, oncologists need to be aware of the rare possibility that patients on ICI may manifest signs and symptoms attributable to dysfunction of the hypothalamic–pituitary axis. This knowledge is essential in order to preserve cancer patients from complications due to late diagnosis or misdiagnosis. Indeed, if promptly suspected, the hypothalamic–pituitary dysfunction may be successfully treated once endocrinological consultation is rapidly requested and provided.

**Abstract:**

Immune checkpoint inhibitors (ICI) prolong the survival in an increasing number of patients affected by several malignancies, but at the cost of new toxicities related to their mechanisms of action, autoimmunity. Endocrine toxicity frequently occurs in patients on ICI, but endocrine dysfunctions differ based on the ICI-subclass, as follows: agents targeting the CTLA4-receptor often induce hypophysitis and rarely thyroid dysfunction, which is the opposite for agents targeting the PD-1/PD-L1 axis. Recently, few cases of central diabetes insipidus have been reported as an adverse event induced by both ICI-subclasses, either in the context of anterior hypophysitis or as selective damage to the posterior pituitary or in the context of hypothalamitis. These new occurrences demonstrate, for the first time, that ICI-induced autoimmunity may involve any tract of the hypothalamic–pituitary axis. However, the related pathogenic mechanisms remain to be fully elucidated. Similarly, the data explaining the endocrine system susceptibility to primary and ICI-induced autoimmunity are still scarce. Since ICI clinical indications are expected to expand in the near future, ICI-induced autoimmunity to the hypothalamic–pituitary axis presents as a unique in vivo model that could help to clarify the pathogenic mechanisms underlying both the dysfunction induced by ICI to the hypothalamus–pituitary axis and primary autoimmune diseases affecting the same axis.

## 1. Introduction

Immune checkpoint inhibitors (ICI) are monoclonal antibodies (mAbs) exerting their anticancer activity by overcoming the host immune tolerance induced by cancer cells [1,2]. ICI have significantly improved survival in an increasing number of patients affected by various solid and hematological malignancies [2]. Unfortunately, together with the clinical achievements, ICI may trigger several side effects, namely, immune-related adverse events (irAEs), that are recognized as due to autoinflammation/autoimmunity in their pathogenesis [3]. ICI-related toxicity may potentially involve any tissue, organ, or apparatus and is mainly unpredictable [4].

Interestingly, for unknown reasons, endocrine glands are among the most frequently affected organs by ICI-induced autoinflammation/autoimmunity [3,4,5,6]. The endocrine toxicity induced by ICI seems to be influenced, among other factors, by the type of ICI, its dose, their combined use, and a previous medical history of autoinflammatory/autoimmune disease [4]. Recently, further factors, including aging, gender, lifestyle, metabolic dysfunctions [5], and intestine microbiome [4], have been suggested to determine the outcome of ICI treatment, but their influence on endocrine toxicity needs to be better understood. Thyroid and pituitary dysfunction are the most frequent endocrinopathies associated with ICI therapy. However, other endocrine glands may be more rarely affected (adrenals, parathyroids, pancreas), and metabolic abnormalities have also been described [7,8]. Recently, central diabetes insipidus (CDI) has been reported as an irAE deriving from the dysfunction of posterior pituitary/hypothalamus in patients who are receiving treatment with ICI [9,10,11,12,13,14,15,16,17,18,19]. Herein, we briefly summarize the current evidence regarding ICI-induced injury to the hypothalamus–pituitary axis.

## 2. The Hypothalamic–Pituitary Axis

**Figure 1:**
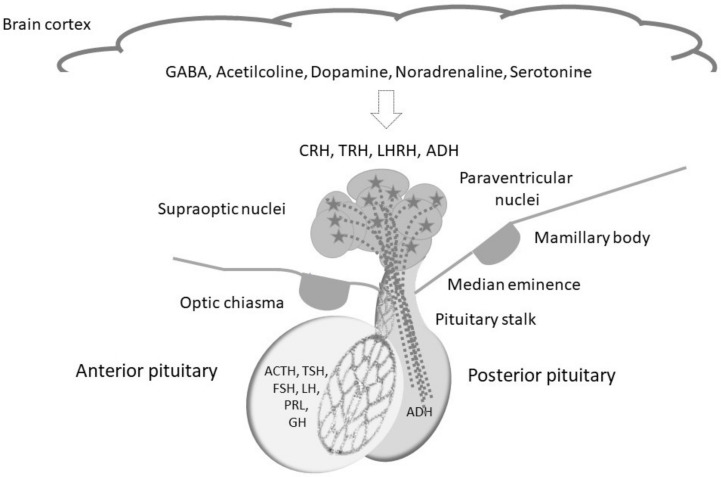
A schematic representation of the hypothalamus-pituitary axis. ACTH, adrenocorticotropic hormone; ADH, anti-diuretic hormone; CRH, corticotropin-releasing hormone; FSH, follicle-stimulating hormone; GABA, gamma-aminobutyric acid; GH, Growth hor-mone; LH, luteinizing hormone; LHRH, luteinizing-releasing hormone; PRL, prolactin; TRH, thyro-tropin releasing hormone; TSH, thyroid-stimulating hormone.

The hypothalamus is a complex brain area located in the median eminence, below the third ventricle, just above the optic chiasm and the pituitary gland (Figure 1). The pituitary gland lies in the sella turcica below the optic chiasm, outside of the dura, and is divided into anterior and posterior lobes (separated by a virtual intermediate area). Notably, their embryogenesis explains the structural and functional differences between the anterior and posterior parts of the pituitary. The anterior lobe derives from the oral ectoderm, while the posterior and hypothalamus have a neuroectodermal origin. The pituitary stalk connects the median eminence to the pituitary gland, passing through an opening in the dura surrounding the brain [20]. 

The hypothalamus acts as the coordinating center of the endocrine system. It merges the signals derived from the upper brain cortex inputs, autonomic function, environmental stimuli (e.g., light and temperature), and peripheral endocrine feedback. In turn, the hypothalamus releases specific signals to the pituitary gland, which then delivers hormones that influence most of the endocrine systems in the body. The hypothalamic–pituitary axis directly regulates the functions of the thyroid, adrenals, and gonads and influences growth, milk production, and water balance [21]. Additionally, the hypothalamus regulates body homeostasis in many vital aspects, including behaviors, metabolisms, and temperature regulation [21,22]. Notably, the posterior lobe of the mature pituitary is composed of hypothalamic axon terminals from the magnocellular neurons of the paraventricular nucleus and supraoptic nucleus [23,24]. The pituitary stalk connects the pituitary and hypothalamus and contains neural and vascular elements. Various disorders may affect the pituitary and the hypothalamus, such as tumors, radiation/surgery, and head trauma, as well as vascular, genetic, infectious, and autoimmune disease, often resulting in hypopituitarism. Additionally, when the hypothalamus is harmed, several functions can be altered, including body temperature regulation, hunger, thirst, sleeping regulation, and behavior (“hypothalamic syndrome”) [20]. Autoimmunity triggered to the hypothalamic–pituitary axis by ICI is an apparent paradox. Monoclonal antibodies, including ICI, are unable to cross the blood-brain barrier, due to their high molecular weight and hydrophilic nature. This should, in theory, prevent the brain from pharmacological interactions with these agents. However, as the capillaries and veins of the portal system of the diencephalic region are fenestrated, several molecules, including drugs that are usually blocked at the blood-brain barrier, can readily cross it.

## 3. Injury to the Pituitary Gland

Hypophysitis is the acute or chronic inflammation of the pituitary gland. It is classified as adenohypophysitis, infundibulo-neuro-hypophysitis, or panhypophysitis, depending on whether the anterior lobe, the posterior lobe, and the pituitary stalk, or the last two, are damaged [25,26,27]. Based on pathology, hypophysitis can be categorized in two more common forms (lymphocytic and granulomatous) and three rarer variants (xanthomatous, necrotizing, and plasma-cell rich) [4,25,26,27]. Another classification is based on the etiology, identifying primary and secondary forms. Primary hypophysitis, the most common form, has autoimmune pathogenesis with inflammation isolated to the pituitary gland and has no causative agent. Secondary hypophysitis is characterized by pituitary inflammation associated with local disorders, systemic diseases, or medications. For local disorders, pituitary inflammation appears as a reaction to a sellar disease (i.e., Rathke’s cleft cyst, craniopharyngioma, germinoma, and pituitary adenoma). In systemic diseases, hypophysitis may stem from infectious diseases or inflammatory disorders (e.g., tuberculosis, syphilis, sarcoidosis, granulomatosis with polyangiitis, or sellar metastases). Treatment-related hypophysitis may complicate surgery and/or radiotherapy or may be a side effect of immunomodulatory drugs [25,26,27,28,29]. In the past years, drug-induced hypophysitis was rarely diagnosed, mainly in interferons or ribavirin treatment [25,26,27]. Recently, with the advent of ICI, hypophysitis has been among the most frequent endocrine irAEs [7,8]. However, a substantial difference exists in the hypophysitis incidence according to the ICI subclass (anti-CTLA4 mAb versus anti-PD1/PDL1 mAb) and the treatment based on a single ICI or an ICI combination. Hypophysitis incidence is 3.2% for anti-CTLA4 mAbs, 0.4% for anti-PD-1 mAbs, and <0.1% for anti-PD-L1 mAbs, when used as a single agent, and 6.4% for combination therapy. Compared to PD-1 monotherapy, hypophysitis more commonly occur during ipilimumab (an anti-CTLA4 mAb) monotherapy (OR, 0.29; 95% CI, 0.18–0.49; *p* < 0.001) than in combination therapy (OR, 2.2; 95% CI, 1.39–3.60; *p* = 0.001) [7]. Interestingly, in contrast to lymphocytic hypophysitis, which is more common in women, ICI-hypophysitis has been reported two to five times more frequently in men (particularly over 60 years old) than in women [28,29]. Moreover, the AE seemed not to be more frequent in any cancer subtype [7,8].

## 4. Anterior/Adeno-Hypophysistis: Clinical Aspects

Patients with anterior hypophysitis can present with a wide variety of symptoms, including the following: weakness, fatigue, headache, visual impairment, anorexia, confusion, memory loss, loss of libido and erectile dysfunction, labile mood, insomnia, temperature intolerance, subjective sensation of fever, and chills [5,25,27]. However, in patients with ICI-induced hypophysitis, visual impairment rarely occurs, as the enlargement of the anterior pituitary is rarely so marked that it exerts a mass effect on the optic chiasm [29]. Contrast-enhanced MRI may show some enlargement of the pituitary gland, sometimes with a thickening of the hypophyseal stalk or a normal-size gland. In some cases, the pituitary gland enhances homogeneously, whereas, in other cases, there is a heterogeneous enhancement [25,27]. However, the entity and quality of pituitary enlargement may depend on the timing at which the MRI is taken, as the enlargement may be transient and may precede the clinical diagnosis of hypophysitis. The pituitary hormones are variably altered, with highly frequent central adrenal insufficiency, central hypothyroidism, and less frequent hypogonadotropic hypogonadism [4,25,26,27]. Alterations in prolactin levels are uncommon, while a deficit in vasopressin (central diabetes insipidus, CDI) is present in 17–48% of patients who are diagnosed with primary autoimmune hypophysitis [27,30]. Notably, hypophysitis triggered by anti-CTLA-4 mAbs (ipilimumab, tremelimumab) often leads to pan-hypopituitarism and is associated with mild pituitary enlargement. In contrast, hypophysitis induced by anti-PD-1 mAbs (nivolumab, pembrolizumab) or anti-PD-L1 mAbs (avelumab, atezolizumab, durvalumab) may present with isolated and severe ACTH deficiency and no mass effect symptoms [29]. Cases of polyendocrine syndrome have also been reported [31].

## 5. Injury to the Posterior Pituitary: Clinical Aspects

An injury to the posterior pituitary may almost invariably cause CDI due to the vasopressin secretion deficit in blood circulation. Vasopressin (antidiuretic hormone, ADH) is secreted by specific hypothalamic neurons (see below) and is stored in the posterior pituitary. Therefore, CDI results from vasopressin deficiency due to hypothalamic (see below) or pituitary disorder. Most CDI cases are idiopathic [20,32,33] or result from primary cancers [32,33], metastatic lesions [34,35], or infiltrative diseases (i.e., Langerhans cell histiocytosis) [20,35]. Notably, in many idiopathic cases, autoimmune pathogenesis is suggested based on detecting anti-vasopressin antibodies [20,32,33,34,35,36]. CDI may also be caused, less frequently, by genetic disorders [20,32,33,35], neurosurgery [32], trauma [33], or hypoxic encephalopathy [20,32,35]. Among anticancer drugs, temozolomide may rarely cause CDI [37,38,39]. Typical symptoms of patients with CDI include polyuria, nocturia, and polydipsia, due to the initial elevation in serum sodium and osmolality [20,32,33,34,35,36]. In the case of underlying neurologic diseases, neurologic symptoms may also be present [20,34]. In patients with untreated CDI, the serum sodium concentration is often high or in the high normal range, which stimulates thirst to compensate for the urinary water losses [20,32,33,34,35,40]. If thirst is impaired or the patient is not autonomous in drinking, moderate-to-severe hypernatremia can develop [20,32,33,34,35]. Moreover, CDI symptoms may vary according to whether the impairment affects one or more of the hypothalamic-posterior pituitary sites involved in ADH secretion (i.e., the hypothalamic osmoreceptors, the supraoptic or paraventricular nuclei, or the superior portion of the supra-opticohypophyseal tract) [20,32,33,34,35]. In particular, if the damage only involves the tract below the median eminence or the posterior pituitary, usually only transient polyuria occurs because the ADH produced in the hypothalamus can still be secreted into the systemic circulation via the portal capillaries in the median eminence [20,32,33,34,35]. In persistent CDI, the MRI high-signal intensity of the posterior pituitary may be absent due to the failure to synthesize, transport, or store ADH granules, being as the posterior pituitary and pituitary stalk are homogeneously enhanced following the administration of contrast enhancement [41]. The diagnosis of CDI is based on the polyuria-polydipsia syndrome, on serum/plasma and urine studies, the response to exogenous vasopressin, and being an absent response suggestive of nephrogenic diabetes insipidus [20,32,33,34,35,36]. A water deprivation test (showing failure to concentrate urine maximally) or an assessment of ADH serum levels may be required in order to confirm the CDI diagnosis [34,42]. Recently, the evaluation of serum copeptin has been proposed in order to improve the accuracy of CDI diagnosis [42]. CDI treatment requires desmopressin or lypressin [42,43,44,45].

## 6. Injury to the Hypothalamus: Clinical Aspects

In the case of hypothalamic injury, the clinical syndrome will depend on the size and extent of the affecting lesion. If it is minimal and only involves specific hypothalamic nuclei, discrete symptoms may be registered; on the contrary, larger lesions, which are more frequent, present with multiple dysfunctions [46]. Adults with hypothalamic dysfunction can present with the following symptoms: fatigue, weakness, nausea or vomiting, visual disturbances (i.e., worsening of visual acuity, blurred vision, diplopia, defect of the oculomotor nerve), disturbances in appetite and sleep, disturbance of autonomic regulation, dementia, and hormonal deficiencies. The endocrine abnormalities that are seen in hypothalamic syndromes usually result in pituitary hyposecretion, due to hypothalamic releasing hormones and ADH deficiencies. However, due to the loss of inhibitory hormones (dopamine), hypersecretion can also occur (hyperprolactinemia) [46]. Causes of hypothalamic damage, particularly the anterior hypothalamus, include craniopharyngiomas and optic nerve gliomas [22,46]. However, radiation [22,47], surgery [22,48], head trauma [46], infectious diseases [46,47], paraneoplastic syndromes [49,50], and genetic disorders [22,46,47], may also affect the hypothalamus. It seems to occur more frequently in female patients and is associated with other autoimmune disorders, such as rheumatoid arthritis and Hashimoto thyroiditis [49]. The peak of incidence is around 40 years, similar to that reported in patients who are affected by hypophysitis [46,47,48]. The pathogenic mechanisms of hypothalamitis are not entirely known, being however considered an autoimmune disease [22,46,47,48,49,50]. Hypothalamitis has been described as a subtype of autoimmune (lymphocytic) hypophysitis; however, some cases of isolated hypothalamic involvement with no inflammatory lesions in either the pituitary gland or infundibulum were reported [22,46,47,48,51]. It is still a topic of debate if autoimmune hypothalamitis may derive from an extension to the hypothalamus of an autoimmune hypophysitis [47,48] or vice versa [22]. Hypothalamitis can be classified (Table 1) as primary and secondary to autoimmune hypophysitis, inflammatory diseases (e.g., basilar meningitis and granuloma, sarcoidosis, histiocytosis, sphenoid osteomyelitis, eosinophilic granuloma), infectious diseases (e.g., herpes simplex virus, cytomegalovirus, human herpesvirus 6–7, West Nile virus, Japanese encephalitis, tuberculosis, listeria, Lyme disease, toxoplasmosis, acute disseminated encephalomyelitis, etc.), paraneoplastic limbic encephalitis (from small cell lung carcinoma, testicular teratoma, breast carcinoma, and Hodgkin’s lymphoma) [22,46,47]. Recently, Tsuma et al. [13] reported the unique case of hypothalamitis secondary to atezolizumab, an anti-PD-L1 mAb, in a patient affected by metastatic bladder cancer (see below). The diagnosis and management of hypothalamitis are challenging due to the rarity of the disease. Moreover, it has been speculated that posterior pituitary inflammation might be a component of hypothalamitis [22,52]. Notably, the diagnosis requires a high level of suspicion by clinicians. The main symptoms prompting clinical suspicion include partial or panhypopituitarism (at various degrees), neuropsychiatric and behavioral disorders, and autonomic and metabolic regulation disturbances. MRI is essential in diagnosing hypothalamus injuries (Table 2); however, it can be normal in many cases, especially at the early onset of symptoms [41,51]. The therapeutic management includes the following three main aspects: treatment of the causative condition, immunosuppressive therapy (once an infectious disease has been excluded), and hormonal replacement therapy, as needed [22,46,47].

## 7. ICI-Induced Injury to the Posterior Pituitary and Hypothalamus

Patients treated with ICI rarely present CDI as an autoimmune syndrome deriving from an injury to the posterior pituitary/hypothalamus. According to data from the WHO global database of individual case safety reports [53], between January 2011 and March 2019, a total of 6089 ICI-related endocrine AEs were reported, of which 1144 (18.8%) were pituitary events, including hypophysitis (835 reports), hypopituitarism (268 reports), pituitary enlargement (28), and other (13), while CDI was reported in 7 out of 1072 (0.7%) of the registered hypophysitis/hypopituitarism cases. Currently, the only clinical features of the damage induced to the posterior pituitary–hypothalamic axis by ICI are derived from 11 case reports that are available in the current literature (summarized in Table 2) [9,10,11,12,13,14,15,16,17,18,19]. All of the patients, except two, were affected by solid malignancies (*N* = 3, melanoma; *N* = 1, prostate cancer; *N* = 1, Merkel cell carcinoma; *N* = 1, non-small cell lung cancer; *N* = 1, pleural mesothelioma; *N* = 1 hypopharynx cancer; *N* = 1, bladder cancer); 10/11 (91%) patients were male, and the median age was 62. Polyuria was reported as the presenting symptom in 9/11 (82%) patients, then diagnosed with CDI. CDI resulted from ICI-induced panhypophysitis in five patients [9,10,15,16]; in a further four patients [12,14,17,18], it was attributed to isolated damage to the posterior pituitary; in one case, CDI derived from hypothalamitis (13), while in another [19] nivolumab (an anti-PD1 mAb) seemed to trigger infundibulo-neurohypophysis, whereas an anterohypophyseal metastasis appeared to cause anterior hypopituitarism. Interestingly, anti-CTLA4 mAbs (ipilimumab or tremelimumab), alone or anti-CTLA4 mAb with anti-PD1/PDL1 mAb combinations, were the causative agents in the cases of panhypophysitis, while isolated CDI was related to treatment with an anti-PD1/PD-L1 mAb. In the patient affected with hypothalamitis, atezolizumab was considered to be the causative agent.

## 8. Discussion

ICI are revolutionary anticancer mAbs acting by renewing antitumor activity of the host immune system escaped by cancer cells through the negative modulation of some receptors expressed on T cells, called immune checkpoints [1,2]. These receptors, once activated, prevent local and systemic excess of the normal immune system response, being dysregulated in autoimmune conditions. CTLA4, PD1, and its ligand-1 and -2, are among the most studied immune checkpoints and are the main targets of the available ICI [1,2]. Therefore, the pharmacological blockade of immune checkpoints can result in the reactivation of antitumor immunity and autoinflammation/autoimmunity at sites different from cancer, which clinically manifests as irAEs prevalently involving the skin, gastrointestinal apparatus, and the endocrine system [3]. Hormone treatment inherently interferes with the endocrine functions aiming at therapeutical goals. On the contrary, anticancer drugs infrequently injure the endocrine glands [54]. Among exceptions, immune modulators (i.e., interferons and interleukin-2) and tyrosine kinase inhibitors may cause thyroid dysfunction, and cytotoxic agents may variably affect ovarian function [55,56,57,58]. However, only immune modulators claim alterations in the immune system among their toxicity mechanisms, including the overproduction of cytokines and, indirectly, the activation of both T cells by some cytokines (i.e., IL-1) and the pathways shared with the neuroendocrine system [54,55,59]. Similarly, ICI-related endocrine toxicity is attributed to autoinflammation/autoimmunity unleashed in the endocrine glands (pituitary, thyroid, adrenals, etc.) [4,6]. Interestingly, since the appearance of ICI, the spectrum of the endocrine toxicities to the hypothalamic–hypophysis axis has widened. Recently, a few cases of CDI have been reported in patients under ICI, occurring not only in the context of panhypophisitis but also as an isolated side effect, resulting from selective damage to the posterior pituitary [9,10,11,12,13,14,15,16,17,18,19]. Interestingly, hypothalamitis was diagnosed in a patient on treatment with atezolizumab, an anti-PD-L1 mAb [13]. These reports add a relevant contribution to defining the clinical spectrum of hypothalamus–pituitary autoimmunity induced by ICI. Indeed, only anterior hypophysitis was initially reported in patients who were treated with anti-CTLA4-mAbs (ipilimumab, 1–19%) and less frequently in patients on treatment with anti-PD1/PD-L1 mAbs (≈1%) [7,8]. Currently, the spectrum of ICI-induced hypothalamus–pituitary autoimmunity includes anterior hypophysitis (AH), infundibulo-neurohypophysis (INH), panhypophysitis (PH), and hypothalamitis (HT). Remarkably, the relative incidence of each condition induced by ICI resembles the epidemiology of primary autoimmune hypothalamic–pituitary dysfunctions, showing in their rarity the following order of frequency: AH > INH > HT. However, the pathophysiology regarding the higher prevalence of anterior hypophysitis than posterior pituitary or hypothalamus involvement by primary or ICI-induced disease remains unexplained.

### 8.1. Pathogenic Aspects

Pathogenetically, the initial evidence suggests that various mechanisms may contribute to the onset of the hypothalamic–pituitary dysfunction in patients on treatment with ICI, including the expression of molecules acting as immune checkpoints (i.e., CTLA-4, PD-1/PD-L1), distinct types of hypersensitivity triggered by different ICI-subclasses, activation of IL17 pathway, and some CTLA-4 gene polymorphisms. Regarding ICI-induced anterior hypophysitis, Iwama et al. [60] demonstrated that CTLA4 is expressed on pituitary cells, suggesting a potential role of the receptor in the pituitary damage triggered by anti-CTLA4-mAbs. Very recently, Okabe et al. [61] reported the results obtained from the first autopsy case of hypophysitis induced by nivolumab. Pathologically, lymphocytes (predominantly CD8-positive T cells) infiltrated the anterior pituitary, and CD68-positive macrophages and CD20-positive B-cells were also detected. The number of pituitary cells, especially ACTH-positive cells, decreased, but necrosis and fibrosis were absent. Notably, some pituitary cells expressed PD-L1. IgG and C4d were deposited on pituitary cells, but IgG4 (the subclass of nivolumab) was not detected [61]. These findings indicate that type I and type II hypersensitivity mechanisms may concur to trigger the hypophysitis induced by anti-PD1 mAbs and that the inflammatory mechanisms underlying hypophysitis caused by anti-PD1 and anti-CTLA4 mAbs may be different. These data let us hypothesize that the prevalent expression of a specific type of immune checkpoint in an endocrine gland may justify the prevalent toxicity attributable to an ICI-subclass. Indeed, it is now clear that endocrine toxicity in patients receiving ICI(s) depends on the mAb(s) received. The first ICI clinically available, ipilimumab, was associated with a high variable rate of anterior hypophysitis and a low rate of thyroid dysfunction. However, with the clinical availability of anti-PD1/PD-L1 mAbs, the prevalence of those endocrine toxicities has reversed. Compared with ipilimumab, PD-1 inhibitors are associated with a significantly greater risk of hypothyroidism (OR, 1.89; 95% CI, 1.17–3.05; *p* = 0.03). Moreover, the risk of hyperthyroidism was higher with PD-1 versus PD-L1 inhibitors (OR, 5.36; 95% CI, 2.04–14.08; *p* = 0.002) [7]. Interestingly, ICI-induced hypophysitis shows some distinctive clinical features from primary hypophysitis, including higher prevalence in males, older age at diagnosis, earlier time onset, severe hypocortisolism, and often persistent hypopituitarism, while the posterior pituitary and optic chiasm are rarely involved [29]. ICI-induced hypophysitis is also different when caused by CTLA-4 blockade or PD-1/PD-L1 blockade. Anti-CTLA-4-induced hypophysitis often leads to pan-hypopituitarism and is associated with mild pituitary enlargement. Instead, hypophysitis induced by PD-1/PD-L1 blockade is usually characterized by isolated and severe ACTH deficiency, rarely mass effect symptoms, and no imaging abnormalities [29]. Those differences may again correspond to different mechanisms of toxicity. It has been suggested that the differences in ICI structure may explain the different rates of pituitary toxicity among ICI. Indeed, IgG subclasses variably activate antibody-dependent cellular cytotoxicity (ADCC) and the classical complement cascade, with IgG1 exerting more potent effects than IgG2 and IgG4 subclasses, while IgG4 cannot activate the classical complement cascade [62]. Among anti-CTLA4 mAbs, ipilimumab is an IgG1 mAb, while tremelimumab is an IgG2 mAb. Among anti-PD1/PD-L1 mAbs, avelumab, durvalumab, and atezolizumab are IgG1 mAbs, while nivolumab, pembrolizumab, and sintilimab are IgG4 mAbs [6,62]. These aspects may support the higher incidence of hypophysitis in patients on ipilimumab (IgG1) than on anti-PD1/PD-L1 mAbs. Recently, the involvement of pro-inflammatory Th1, Th17, and Th1/17 subsets has been suggested in patients who are affected by autoimmune and ICI-induced hypophysitis [63,64]. In a mouse model of primary hypophysitis, Chalan et al. [63] identified CD4+T lymphocytes displaying a Th17 and Th1/Th17 phenotype as the main pituitary-infiltrating immune cell population. Moreover, significantly higher IL-17A, CD4, and class II MHC transactivator mRNA levels were found in the pituitary glands of patients diagnosed with primary hypophysitis compared with adenoma and normal gland. All of the patients (*n* = 3) affected by secondary hypophysitis showed detectable IL-17A levels, while other cytokines were not dosable in their pituitaries. Levels of IFN-γ, IL-4, IL-10, and TGF-β did not differ among the groups [63]. Consistent data was seen in a patient affected by mesothelioma who developed fatal neuroendocrine toxicity (insulin-dependent diabetes, hypophysitis, and a myasthenia-like syndrome) while he was on treatment with a dual mAb blocking PD1 and TIM3 [64]. Elevated levels of interleukin 17, but no other cytokines, were identified in the patient’s plasma, but not in the controls (healthy volunteers and patients treated with immunotherapy who did not complain of neuroendocrine toxicities) [64]. However, despite these findings, the pathogenic role of the IL-17 pathway in primary and ICI-induced pituitary (and hypothalamic) dysfunction needs to be better clarified. Finally, a potential role of some gene polymorphisms in prompting ICI-related toxicity has been suggested [6,65]. Some CTLA-4 polymorphisms are commonly associated with autoimmune disorders, including endocrinopathies (i.e., T1DM, Graves’ disease, autoimmune hypothyroidism, and Addison’s disease) [6,66]. Only in a few studies has a linkage between PD-1 polymorphisms and T1DM has been suggested, while PD-L1 polymorphisms were found to be linked to T1DM, Graves’ disease, and Addison’s disease [6,66]. Based on these data and the well-documented association between some germline genetic variants and adverse events induced by anticancer agents [67], an association between specific gene polymorphisms of immune checkpoints and the development of ICI-induced toxicities has been postulated [6,65]. However, studies on the role of gene polymorphism in ICI-induced hypothalamic–pituitary dysfunction are needed. The role of autoimmunity in ICI-induced (anterior) hypophysitis is also sustained by detecting autoantibodies against cells secreting TSH, FSH, and ACTH in patients with anti-CTLA4-induced hypophysitis [68]. Several studies have explored the role of anti-pituitary (APA) and anti-hypothalamic antibodies (AHA) in primary (lymphocytic) hypophysitis [27,69,70,71,72] but not in cancer patients on ICI treatment. Interestingly, APA were detected in two out of four patients presenting with anti-PD1/PD-L1 related hypophysitis [73]. Very recently, Bellastella et al. [74] reported the first cross-sectional study on APA and AHA antibodies in patients treated with anti-PD1/anti-PD-L1 mAbs compared with healthy controls, demonstrating for those antibodies a role in hypothalamic–pituitary toxicity triggered by anti-PD1/PD-L1 mAbs. A higher prevalence of APA and AHA was observed in ICI-treated patients compared to controls; in detail, after nine weeks of ICI treatment, 7 out of 13 patients became APA-positive, and three became AHA-positive, also showing an increase in prolactin and a decrease in ACTH and IGF-1 levels compared with basal values. However, the role of APA and AHA in patients on treatment with anti-CTLA4 mAbs remains to be evaluated. If some findings provide an early contribution to clarify mechanisms of ICI-induced damage to the anterior pituitary, very few experimental data are available about the pathogenesis of ICI-induced injury to the posterior pituitary/hypothalamus. Recently, Iervasi et al. [75], for the first time, demonstrated the expression of the PD-L1 on the hypothalamic cells of a primate, providing the basis for a potential explanation for the onset of a destructive inflammatory hypothalamic mass during treatment with atezolizumab, as reported by Tshuma et al. [13]. Interestingly, indirect comparison of data from clinical trials on ICI might suggest that atezolizumab could have a sort of toxicity tropism for the central nervous system (CNS), based on the higher rate of atezolizumab-related CNS toxicity compared with other ICI (i.e., immune-related encephalitis, 0.75% vs. 0.12%, respectively) [76]. However, head-to-head data are needed to confirm these speculative suggestions. Interestingly, certain lifestyles, metabolic disorders, and even socio-demographic factors are suggested to modulate the ICI response in patients who are affected by various malignancies [5]. In detail, exercise and diet seemed to improve ICI responsiveness, while alcohol consumption reduced their therapeutic effect by decreasing the mutational burden and hampering antigen presentation by dendritic cells [5]. Obesity was suggested to enhance PD-1 expression, while diabetes and hypertension were consequences of ICI treatment [5]. Among the sociologic factors, sex and race positively influenced the ICI effectiveness because of increased effector T cell activity and PD-1 expression [3,4,5]. On the contrary, aging is suggested to impair ICI response by decreasing functional T cells and increased toxicity [4,5]. An example of a synergistic effect of these factors was observed for obesity and gender, in which obese males showed the most significant impact of ICI [5]. Those factors probably also have a role in triggering ICI-induced endocrine toxicity, including hypothalamic–pituitary dysfunctions, but confirmative studies are awaited.

### 8.2. Clinical Aspects

CDI is the main presenting syndrome in posterior pituitary and hypothalamic primary autoimmunity, and recently in the case of ICI-induced damage to the posterior pituitary–hypothalamus. However, the paucity of related data hinders drawing a definite clinical picture of the latter condition. Furthermore, in the few case reports of ICI-induced CDI, the presence of concomitant symptoms, the workup that led to the diagnosis, the treatment of ICI-induced CDI, and the decision making about maintenance/withdrawal of ICI(s) were highly variable. Nevertheless, some aspects appear worthy of interest. In particular, anti-CTLA4 mAbs seemed to trigger CDI only in the context of panhypophysitis, while anti-PD1/PD-1L mAbs caused an isolated CDI. In other words, in three out of the four patients who developed an anterior hypophysitis, presumably, the inflammatory enlargement of the anterior part of the gland might have involved the posterior pituitary [9,10,11,15]. Consistently, CDI was transient in those patients, resolving concomitantly with the regression of hypophysitis. In the fourth case, CDI occurred two weeks after the onset of an anterior hypophysitis in a patient on a combined treatment of ipilimumab and nivolumab [11]. In this case, it might be speculated that CDI was caused by nivolumab rather than ipilimumab, which probably had already triggered anterior hypophysitis that was resolving when CDI occurred. Accordingly, the ADH deficit was permanent, requiring desmopressin maintenance (data on late follow-up unavailable). In the only case of ICI-induced hypothalamitis, the ADH deficit seemed to be permanent [13].

It would be interesting to draw the differences between primary/autoimmune and ICI-induced hypothalamitis. However, the anecdotal occurrence of hypothalamitis secondary to ICI precludes any speculation. To date, only one case of ICI-induced hypothalamitis and a few cases of primary ones have been described as well. Nevertheless, based on the limited data concerning both of the conditions, some considerations arise that can be worthy of interest in clinical practice and future research. In the case of the ICI-induced hypothalamitis reported by Thsuma et al. [13], a biopsy of the hypothalamic mass was not considered feasible, and no evaluation of the pituitary autoimmunity was made. Histopathological data are always crucial to characterizing a disease, especially if rare; however, a hypothalamic biopsy may be difficult to obtain, and clinical and ethical issues may contraindicate it. Some studies suggest a role for AHA in rare cases of autoimmune hypothalamitis [13]. To our best knowledge, the initial data on hypothalamic/pituitary antibodies in patients treated with anti-PD1/PD-L1 mAbs are available from only one cross-sectional study [74]. However, no case of ICI-induced hypothalamitis was included in that study, and serum autoimmunity was not assessed in the unique case of ICI-induced hypothalamitis. Therefore, these antibodies’ pathogenic and diagnostic roles in the ICI-induced hypothalamic/pituitary dysfunction merits more extensive studies. Moreover, since the data explaining the endocrine system susceptibility to autoimmunity remains to be fully elucidated, ICI-induced autoimmunity to the hypothalamic–pituitary axis may be exploited as a unique in vivo model that could help to clarify pathogenic mechanisms underlying the primary autoimmune diseases affecting this endocrine axis.

### 8.3. Management Considerations

Clinically, the accurate diagnosis of the condition causing CDI is, per se, important for obvious reasons. However, in ICI-induced posterior pituitary-hypothalamitis, defining the anatomical site that is damaged by the drug is even more essential in order to choose the appropriate treatment. Indeed, corticosteroids are not recommended in patients who are diagnosed with autoimmune CDI due to posterior pituitary damage, except when pituitary enlargement causes symptoms due to “mass effect” [35,42]. On the contrary, corticosteroids are recommended in primary autoimmune hypothalamitis [22,47]. This treatment was also used in the unique case of ICI-induced hypothalamitis (similar to most cases of hypothalamitis) [13]. Notably, in the sporadic hypothetical case of ICI-induced CDI caused by an occult (in development) hypothalamitis, the patient could not timely receive the appropriate treatment. However, at the moment, the early differentiation of whether CDI derives from either an injury to the posterior pituitary or the hypothalamus remains to be a challenging task. Autoimmune hypothalamitis almost invariably presents with CDI symptoms, but the diagnosis of the underlying condition may be revealed years later. This happened in the only case of ICI-induced hypothalamitis as follows: the patient presented with CDI symptoms approximately eight months before the hypothalamic syndrome, a time frame highly delayed compared to the onset of ICI-induced CDI reported in the available cases (mean: 3.4 months). Unfortunately, MRI cannot be helpful for an earlier differential diagnosis of posterior pituitary damage versus hypothalamitis. Indeed, MRI may frequently be normal in the first phase of hypothalamitis, similar to the many cases of primary CDI (including ICI-induced CDI). Waiting for clinical and translational studies on ICI-induced pituitary–hypothalamus autoimmunity, in the current clinical practice, the diagnosis of ICI-induced hypothalamitis should be based on the criteria suggested by Türe et al. [22] (Table 3). Since CDI was the first clinical manifestation of ICI-induced hypothalamitis, an accurate clinical and radiological follow-up is recommended in patients presenting with CDI [32,33,34,35,41]. On the other hand, fatigue, weakness, nausea, or vomiting, are among the most frequent symptoms in patients diagnosed with hypothalamitis, but they are also common and multifactorial in cancer patients. Therefore, hypothalamitis may be underdiagnosed in patients on treatment with ICI(s). Consequently, the persistence of those symptoms, together with the onset of those classified as “hypothalamic syndrome”, particularly in patients diagnosed with CDI, should prompt oncologists to an early endocrinological and neurological consultation and MRI assessment.

**Figure 2:**
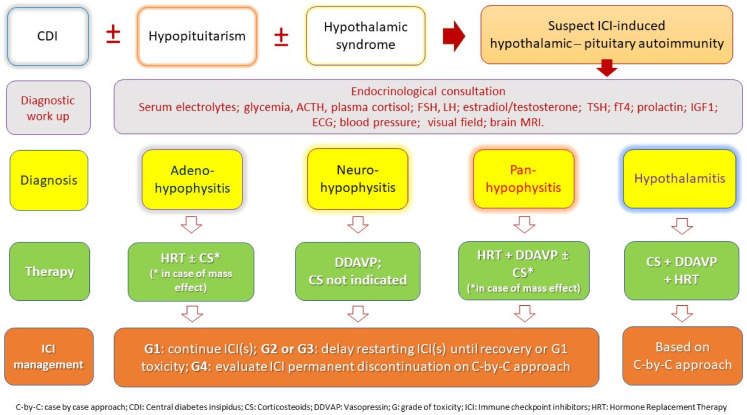
Suggested diagnostic work-up and therapeutic management in the case of suspected ICI-induced hypothalamic–pituitary autoimmunity.

The management of ICI-induced anterior hypophysitis should follow the current guidelines [77,78,79,80,81] and requires the prompt administration of high-dose corticosteroids (e.g., 1 mg/kg/day) in case of symptoms due to optic chiasm compression or “mass effect”. This therapy should be continued for one to two weeks, or until symptom resolution, then rapidly tapered down to physiologic replacement dose [77,78,79,80,81]. Moreover, hormone deficits need to be accurately replaced. Notably, most patients necessitate long-term hormone replacement therapy [77,78,79,80,81]. ICI(s) should be suspended in any grade ≥2 hypophysitis, and endocrinological consultation is required in order to prescribe the appropriate replacement therapy based on the diagnosis of partial or complete hypopituitarism (Figure 2). Once the patient recovers from symptoms, ICI can be restarted in most cases. Moreover, patients on hydrocortisone therapy should have a medical alert device (e.g., alert bracelet) [77,80,81]. In parallel, both the patients and the caregivers should be educated on the possible need for “stress doses” of steroids in certain conditions, such as severe illness, surgery, and infection, and on the emergency use of parenteral dexamethasone or hydrocortisone [77,78,79,80,81].

On the contrary, the management of ICI-induced posterior pituitary-hypothalamic dysfunction can now be only based on the current management of similar conditions, i.e., the primary autoimmune diseases of the posterior pituitary–hypothalamus [32,33,34,35,42]. In detail, in the case of CDI deriving from ICI-induced damage to the posterior pituitary, corticosteroids can be avoided, becoming otherwise essential in the case of CDI deriving from ICI-induced hypothalamitis (Figure 2) and in the case of optical chiasma compression. In any case, CDI-related symptoms (polyuria, dehydration, and hypernatremia) require vasopressin titration and adequate fluid supplementation, according to the grade of toxicity (Figure 2). Similarly, the ICI(s) can be delayed/restarted or permanently discontinued according to the toxicity level of ICI-induced CDI. Whereas, if ICI-induced hypothalamitis is diagnosed, ICI(s) should be temporarily suspended, prioritizing corticosteroid treatment and replacement therapy of endocrine deficits (i.e., ADH and others). Nowadays, the possibility of restarting ICI is a multidisciplinary decision that can be addressed on a case-by-case approach (Figure 2). Notably, a careful clinical and radiological (MRI) program should be organized in order to follow up with patients, in parallel with monitoring cancer response to ICI(s).

Finally, it has been suggested that the development of the pituitary [82,83] or thyroid [84,85] dysfunction is associated with better outcomes after ICI treatment [86,87]. However, the paucity of cases and the absence of data regarding their follow-up preclude any prognostic correlation in patients who are diagnosed with ICI-induced posterior pituitary or hypothalamic dysfunction.

## 9. Future Directions

ICI are expected to widen the therapeutic armamentarium for an increasing number of malignancies in the near future. However, the optimized use of these agents requires reliable tools in order to predict treatment efficacy and toxicity. Currently, several biological factors affecting ICI efficacy have been detected, including tumor mutational burden, the composition of the tumor microenvironment, pathways modulating the expression of the ICI target(s), T-cytotoxic cell infiltration, and other immune cell processes. Efforts are ongoing to test those factors as reliable biomarkers capable of personalizing ICI treatment in clinical practice. Similar studies evaluating the potential biomarkers of toxicity induced by ICI are not available. Recently, sociological factors (e.g., age, gender, race), lifestyle, and metabolic disorders (e.g., sedentariness, alcohol consumption, obesity) have been suggested to, directly and indirectly, modulate the ICI therapeutic efficacy and toxicity. Future prospective studies should correlate not only biological, genetic, and molecular data, but also demographic, lifestyle, and metabolic disorders with ICI response and toxicity, in order to provide clinicians with reliable predictive tools to select the proper treatment for the individual patient. It is currently unknown whether all of the above factors may have a role in the onset of endocrine toxicity induced by ICI, including autoimmunity to the hypothalamic–pituitary axis. Limited to this AE, the prospective assessment of the hypothalamic–pituitary autoimmunity (i.e., anti-pituitary and anti-hypothalamus antibodies and other antibodies) will provide essential data that would contribute to defining its role in early diagnosing potential life-threatening conditions and preventing endocrine insufficiencies requiring life-long replacement therapies. Regarding the management of ICI-induced hypothalamic–pituitary axis dysfunctions, research is needed on the role of corticosteroids in the acute treatment of the injury and their effects on both the outcome and the resolution of ICI-induced endocrine dysfunction and on oncologic response.

## 10. Conclusions

With the reporting of ICI-induced hypothalamitis, ICI have been demonstrated to be a potential threat to each tract of the hypothalamic–pituitary axis. ICI may rarely unleash the posterior pituitary and hypothalamus autoimmunity, but this irAE and the deriving endocrine dysfunctions are expected to become more frequent with the expanding clinical indications of these drugs. According to the current data, the hypothalamus and the pituitary gland seem to be damaged by ICI, with a frequency comparable to primary autoimmunity in those organs. This aspect, and some recent pathological and serological findings, render the ICI-induced autoimmunity an attractive clinical model that might be exploited in order to improve our knowledge about the mechanisms sustaining primary hypothalamic–pituitary autoimmunity. To this aim, future large prospective studies on ICI should incorporate the assessment of the hypothalamic–pituitary autoimmunity, including serum hypothalamic and pituitary autoantibodies, along with other genetic, biomolecular, lifestyle, metabolic, and socio-demographic factors. This comprehensive research might also obtain information about their potential prognostic and predictive role in cancer patients. In the meantime, oncologists should be able to recognize the signs and symptoms of the hypothalamic–pituitary axis dysfunction caused by ICI in cancer patients in order to promptly activate endocrinological consultation, providing them with the appropriate diagnostic workup and treatment early.

## Figures and Tables

**Table 1:** Classifications of the pituitary and hypothalamic diseases.

Classification	Pituitary	Hypothalamus
Anatomical [25,27]	Adenohypophysitis (65%) Infundibulo-neurohypophysitis (10%) Panhypophysitis (25%)	The whole hypothalamus Selective damage to specific hypothalamic nuclei
Pathological [22,25,27,47]	Lymphocytic hypophysitis (68%) Granulomatous hypophysitis (19%) IgG4-related (plasmocytic) hypophysitis (8%) Xanthomatous hypophysitis (4%) Necrotizing hypophysitis (<1%) Mixed forms (lymphogranulomatous; xanthogranulomatous)	-
Pathogenic [22,25,27,46,47,49]	**Primary** Unknown Autoimmune	**Primary** Unknown Autoimmune
	**Secondary** **Local disorder** (Germinoma, Rathke’s cleft cyst, craniopharyngioma, pituitary adenoma, pituitary lymphoma) **Systemic disease** - **Associated with autoimmune diseases** - **Inflammatory** (Wegener’s granulomatosis, Sarcoidosis) - **Infectious** (Mycobacterium tuberculosis, Treponema pallidum, Tropheryma whipplei, Borrelia, Brucella, Cytomegalovirus, Coronavirus, Coxsackie viruses, Enterovirus, Herpes simplex virus, Hantavirus, Influenza viruses, Tick-Borne encephalitis virus, Varicella-zoster virus Aspergillus, Nocardia, Candida albicans, Pneumocystis jirovecii, Toxoplasma gondii) - **Thymoma and other malignancies** (anti-Pit-1 antibody syndrome) - **Metastases** **Sellar and parasellar disease** **Treatment related** (Surgery, radiotherapy, drugs: Ribavirina, Interferon, Ustekinumab, Immune checkpoint inhibitors)	**Secondary** **Local disorder** (Autoimmune hypophisitis, basilar meningitis, sphenoid osteomyelitis, eosinophilic granuloma, craniopharyngioma, germinoma, pituitary adenoma, hypothalamic lymphoma) **Systemic disease** - **Inflammatory** (Wegener’s granulomatosis, Sarcoidosis) - **Infectious** (Mycobacterium tuberculosis, Syphilis, Cytomegalovirus, Herpes simplex virus, Human Papilloma Virus 6–7, West Nile Virus, Japanese encephalitis, Lysteria, Lyme disease, Toxoplasmosis, Acute disseminated encephalitis) - **Metastases** - **Paraneoplastic limbic encephalitis** (due to small cell lung cancer, breast cancer, Hodgkin lymphoma, testicular teratoma) **Treatment related** - Surgery - Radiotherapy - Drugs (atezolizumab, an immune checkpoint inhibitor—anti-PDL1 monoclonal antibody)
Clinical [7,8,25,27,35,46]	**Anterior hypopituitarism** **Central diabetes insipidus** **Anterior hypopituitarism and central diabetes insipidus**	**Central diabetes insipidus** **Neurological disturbance** (bitemporal hemianopsia, blurred vision, apraxia, cognitive dysfunction, hyperpyrexia, memory impairment) **Behavioral disturbance** (hyperphagia, personality change)

**Table 2:** Relevant clinical data obtained from the cases of ICI-induced CDI available in the current literature.

Authors	Age	Sex	Malignancy	Drug(s)	ICI Target/IgG-Subclass	Injury to			Median Time to Onset (Days)	MRI		ICI Delay/Discontinuation (Dis)	GC Treatment	Follow up (Days)
						Anterior Pituitary	Posterior Pituitary	Hypothalamus		Anterior Pituitary	Posterior Pituitary BS			
Dillard et al. [9]	50	M	Prostate	Ipilimumab	CTLA4/IgG1	Yes	Yes	No	84	Normal	evident # --	Normal end (4th cycle)	Yes	NR
Nallapanemi et al. [10]	62	M	Melanoma	Ipilimumab	CTLA4/IgG1	Yes	Yes	No	121	Normal	data NR--	Normal end (4th cycle)	Yes	180
Gunawan et al. [11]	52	M	Melanoma	Ipilimumab + Nivolumab	CTLA4/IgG1 PD-1/IgG4	NR	Yes	No	28	Hemorrhagic	data NR --	Dis	Yes	NR
Zhao et al. [12]	73	M	MCC	Avelumab	PD-L1/IgG1	No	Yes	No	112	Normal	NE --	Dis	No	240
Tshuma et al. [13]	74	F	Bladder	Atezolizumab	PD-L1/IgG1	Yes	No	Yes	270	Normal	Data NR; Hypo-thalamic mass	Dis	Yes	365
Deligiorgi et al. [14]	71	M	NSCLC	Nivolumab	PD-1/IgG4	No	Yes	No	150	Normal	Evident --	Dis	No	0 §
Barnabei et al. [15]	64	M	Melanoma	Ipilimumab	CTLA4/IgG1	Yes	Yes	No	60	Micro-infarcts	Evident --	Delay	Yes	1230
Grami et al. [16]	30	M	AML	Ipilimumab + Nivolumab	CTLA4/IgG1 PD-1/IgG4	Yes	Yes	No	NR	NR	NR	Dis	Yes	NR
Brilli et al. [17]	68	M	Mesothelioma	Tremelimumab + Durvalumab	CTLA4/IgG2 PD-L1/IgG1	No	Yes	No	178	Normal	NE --	Delay	No	570
Yu et al. [18]	60	M	HL	Sintilimab	PD-1/IgG4	No	Yes	No	Immediate	Normal	Nodular signal	Dis	Yes	90
Fosci et al. [19]	62	M	Hypopharynx	Nivolumab	PD-1/IgG4	Yes °	Yes	No	35	Metastasis	NE + stalk enlarged	Dis	Yes	24

ADH, anti-diuretic hormone; AMH, acute myeloid leukemia; GC, glucocorticoids; HL, Hodgkin lymphoma; MCC, Merkel cell carcinoma; MRI, magnetic resonance imaging; ND, not done; NE, not evident; NR, not reported in the paper; NSCLC, non-small cell lung cancer; ° attributed to an anterohypophyseal metastasis. # brain MRI assessment was performed three weeks after the onset of symptoms; § the patient suddenly died, just after the diagnosis.

**Table 3:** Criteria for the diagnosis of hypothalamitis suggested by Türe et al. [22].

Major Criteria
Suprasellar mass Histopathologic findings consisting of autoimmune involvement of the hypothalamus Central diabetes insipidus Partial or complete hypopituitarism Positive anti-hypothalamic antibodies
**Minor Criteria**
Good response of suprasellar mass to immunosuppressive agents Radiologic findings suggesting hypothalamitis on MRI Female gender
